# Gemcitabine as adjuvant chemotherapy in patients with high-risk early breast cancer—results from the randomized phase III SUCCESS-A trial

**DOI:** 10.1186/s13058-020-01348-w

**Published:** 2020-10-23

**Authors:** Amelie de Gregorio, Lothar Häberle, Peter A. Fasching, Volkmar Müller, Iris Schrader, Ralf Lorenz, Helmut Forstbauer, Thomas W. P. Friedl, Emanuel Bauer, Nikolaus de Gregorio, Miriam Deniz, Visnja Fink, Inga Bekes, Ulrich Andergassen, Andreas Schneeweiss, Hans Tesch, Sven Mahner, Sara Y. Brucker, Jens-Uwe Blohmer, Tanja N. Fehm, Georg Heinrich, Krisztian Lato, Matthias W. Beckmann, Brigitte Rack, Wolfgang Janni

**Affiliations:** 1 Department of Gynecology and Obstetrics, Ulm University Hospital, Prittwitzstrasse 43, 89075 Ulm, Germany; 2 Department of Gynecology and Obstetrics, Erlangen University Hospital, Friedrich-Alexander-University of Erlangen-Nuremberg, Comprehensive Cancer Center EMN, Erlangen, Germany; 3 Department of Gynecology and Obstetrics, Biostatistics Unit, Erlangen University Hospital, Friedrich-Alexander-University of Erlangen-Nuremberg, Erlangen, Germany; 4 Department of Gynecology, University Hamburg-Eppendorf, Hamburg, Germany; 5 Gynecologic-Oncological Practice, Hannover, Germany; 6 Gynecologic Practice Dr. Lorenz, N. Hecker, Dr. Kreiss-Sender, Braunschweig, Germany; 7 Hemato-Oncological Practice Dres Forstbauer and Ziske, Troisdorf, Germany; 8 Department of Obstetrics and Gynecology, University Hospital, Ludwig-Maximilians-University of Munich, Munich, Germany; 9 National Center for Tumor Diseases, Division of Gynecologic Oncology and German Cancer Research Center, Heidelberg, Germany; 10 Department of Oncology, Onkologie Bethanien, Frankfurt, Germany; 11 Department of Gynecology and Obstetrics, Tübingen University Hospital, Tübingen, Germany; 12 Department of Gynecology and Breast Center, Charité University Hospital Campus Charité-Mitte, Berlin, Germany; 13 Department of Gynecology and Obstetrics, Düsseldorf University Hospital, Heinrich-Heine University, Düsseldorf, Germany; 14 Department of Gynecologic Oncology, Schwerpunktpraxis für Gynäkologische Onkologie, Fürstenwalde, Germany

**Keywords:** Early breast cancer, Gemcitabine, Chemotherapy

## Abstract

**Background:**

When chemotherapy is indicated in patients with early breast cancer, regimens that contain anthracyclines and taxanes are established standard treatments. Gemcitabine has shown promising effects on the response and prognosis in patients with metastatic breast cancer. The SUCCESS-A trial (NCT02181101) examined the addition of gemcitabine to a standard chemotherapy regimen in high-risk early breast cancer patients.

**Methods:**

A total of 3754 patients with at least one of the following characteristics were randomly assigned to one of the two treatment arms: nodal positivity, tumor grade 3, age ≤ 35 years, tumor larger than 2 cm, or negative hormone receptor status. The treatment arms received either three cycles of 5-fluorouracil, epirubicin, and cyclophosphamide, followed by three cycles of docetaxel (FEC → Doc); or three cycles of FEC followed by three cycles of docetaxel and gemcitabine (FEC → Doc/Gem). The primary study aim was disease-free survival (DFS), and the main secondary objectives were overall survival (OS) and safety.

**Results:**

No differences were observed in the 5-year DFS or OS between FEC → Doc and FEC → Doc/Gem. The hazard ratio was 0.93 (95% CI, 0.78 to 1.12; *P* = 0.47) for DFS and 0.94 (95% CI, 0.74 to 1.19; *P* = 0.60) for OS. For patients treated with FEC → Doc and FEC → Doc/Gem, the 5-year probabilities of DFS were 86.6% and 87.2%, and the 5-year probabilities of OS were 92.8% and 92.5%, respectively.

**Conclusion:**

Adding gemcitabine to a standard chemotherapy does not improve the outcomes in patients with high-risk early breast cancer and should therefore not be included in the adjuvant treatment setting.

**Trial registration:**

Clinicaltrials.gov NCT02181101 and EU Clinical Trials Register EudraCT 2005-000490-21. Registered September 2005.

## Introduction

In research on breast cancer (BC), considerable effort has been put into identifying predictive and prognostic markers to assist in decision-making for or against chemotherapy [1, 2]. However, chemotherapy continues to be one of the main treatment options for patients with unfavorable prognostic factors [3, 4]. During the last few decades, the prognosis for BC patients has been substantially improved through the introduction of chemotherapy using cyclophosphamide, methotrexate, and fluorouracil (CMF), and subsequently the introduction of anthracyclines and taxanes [5]. At the time of recruitment for the SUCCESS-A trial, the sequence of 5-fluoroucacil, epirubicin, and cyclophosphamide (FEC) followed by docetaxel (Doc) was regarded as one of the standard chemotherapy regimens for patients with high-risk early BC, after the regimen had been tested in several studies [6–8], and FEC → Doc showed improved overall survival (OS) in node-positive patients [9]. However, with a 5-year disease-free survival (DFS) probability of approximately 73% treatment for these patients still needed substantial improvement, for instance with the help of active agents from the metastatic setting.

In the metastatic setting, there were promising results for the addition of gemcitabine to taxanes with regard to tumor response, time to progression, and OS [10]. Partial and complete response rates were 26% (95%CI, 21%–32%) in patients receiving paclitaxel monotherapy and 41% (95% CI, 35%–47%) in those treated with addition of gemcitabine (Gem). This benefit contributed to advantages in time to progression and OS [10]. A meta-analysis summarized additional smaller studies and came to the same conclusion: the addition of gemcitabine improves the response and prognosis in first-line treatments, but not for patients receiving later treatment lines [11]. Thus, gemcitabine seemed to be a promising candidate for improvement of adjuvant cytotoxic therapy as it showed good efficacy as a first-line treatment in the metastatic setting and—unlike most other agents—was even associated with an OS benefit [10–13]. Therefore, the aim of the SUCCESS-A trial was to compare efficacy of standard chemotherapy to standard chemotherapy with the addition of gemcitabine in relation to DFS and OS in patients with high-risk early BC.

## Methods

### Study design

SUCCESS-A (NCT02181101) was an open-label, multicenter phase III randomized study and was conducted as an investigator-initiated trial in Germany that enrolled patients ≥ 18 years with an invasive BC and a high recurrence risk, defined as positive lymph nodes, large tumor (pT2/pT3), high tumor grade (G3), negative hormone-receptor status, or young age (≤ 35 years) (see Appendix A for a complete list of inclusion and exclusion criteria). All patients provided written informed consent before entering the study, which was approved by all of the relevant ethics committees in Germany and conducted in accordance with the Declaration of Helsinki.

The SUCCESS-A trial had also an affiliated translational research project with focus on circulating tumor cells, serum tumor marker, and pharmacogenetics [14–17].

### Randomization and treatment

Patients were treated with three cycles of FEC (500/100/500 mg/m^2^) followed by either three cycles of docetaxel (100 mg/m^2^, q3w) (FEC → Doc) or gemcitabine (1000 mg/m^2^ on days 1 and 8) and docetaxel (75 mg/m^2^, q3w) (FEC → Doc/Gem). The randomization ratio was 1:1, and the stratification factors were lymph node status (pN0/pN1/pN2/pN3), hormone-receptor status (negative/positive), tumor grade (G1/G2–G3), menopausal status (pre-/postmenopausal), and HER2 status (negative/positive/unknown). Dose reductions/delays were prespecified as per protocol (Appendix B). There was an additional randomization to treatment with 2 versus 5 years of zoledronate after chemotherapy. Randomization was performed by fax or electronically via internet by the appointed clinical research organization.

### Further and supportive therapies

Antihormonal and HER2 treatment were prespecified as per protocol: premenopausal, HRS-positive women received tamoxifen for 5 years (± goserelin for the first 2 years in women < 40 years), postmenopausal patients only for 2 years, followed by anastrozole for 3 years. HER2-positive patients received trastuzumab for 1 year after chemotherapy completion. The primary surgery had to result in complete resection of the tumor (R0). Axillary surgery was performed in accordance with the national guidelines (either as sentinel node biopsy or lymph node dissection) as was radiotherapy [18, 19].

### End points, follow-up, and data capture

DFS and OS were defined according to the STEEP system [20]. DFS was defined as the period from the date of randomization to the earliest date of disease progression (distant metastasis, local and contralocal recurrence, and secondary primary tumors or death from any cause) or to the last date on which the patient was known to be disease-free (censored). Non-invasive (in situ) cancer events were excluded. OS was defined as the time from randomization to death from any cause or to the last date on which the patient was known to be alive (censored). The maximum observation time was 5.5 years (6 months of chemotherapy followed by 5 years of treatment with zoledronate). For assessment of survival and recurrence, the patients were followed up at the study sites at 3-month intervals for the first 3 years, and every 6 months thereafter.

### Sample size calculation

The sample size calculations were based on a study comparing six cycles of FEC with three cycles of FEC followed by three cycles of docetaxel, which showed that treatment with FEC → Doc would result in a 5-year DFS probability of 78.3% [9]. An improvement by 4% from 78.3 to 82.3% for FEC → Doc/Gem patients was considered to be clinically relevant. On this basis, it was calculated that 743 events would be required to achieve 80% power to show a significant difference in the DFS using the log-rank test and the Wald test in a simple Cox proportional hazards model.

### Statistical methods

The primary objective was to compare the DFS, and the secondary the OS between the two treatment groups (based on the intention-to-treat population). Survival rates were estimated using the Kaplan–Meier product limit method. Simple Cox regression models were fitted to estimate hazard ratios (HRs). For sensitivity analysis, similar analyses were performed for the end points of distant metastasis-free survival and BC-specific survival.

To get HRs adjusted for well-known prognostic factors, a mixed-effects Cox model was fitted with the study center as a random effect and the following predictors as fixed effects: age (continuous), body mass index (BMI; continuous), tumor stage (ordinal; pT1/pT2/pT3/pT4), tumor grade (ordinal; G1/G2/G3), lymph node status (categorical; pN0/pN+), tumor type (categorical; ductal/lobular/other) and receptor status for estrogen, progesterone, and HER2 (each categorical; negative/positive). This model was compared with a mixed-effects Cox model with the same predictors and additionally the treatment arm and its interactions with these predictors using the likelihood ratio test. In case of significance, interaction tests were performed to examine the significance of subgroup-specific variation in treatment effects. Missing predictor values were imputed (median value of continuous or integer predictors, the most common value of categorical or ordinal predictors), and continuous predictors were used as natural cubic spline functions, as done before [21].

All of the tests were two-sided, and a *P* value of < 0.05 was regarded as statistically significant. Calculations were carried out using the R system for statistical computing, version 3.0.1 (R Development Core Team, Vienna, Austria, 2013).

## Results

### Patients

**Fig. 1:**
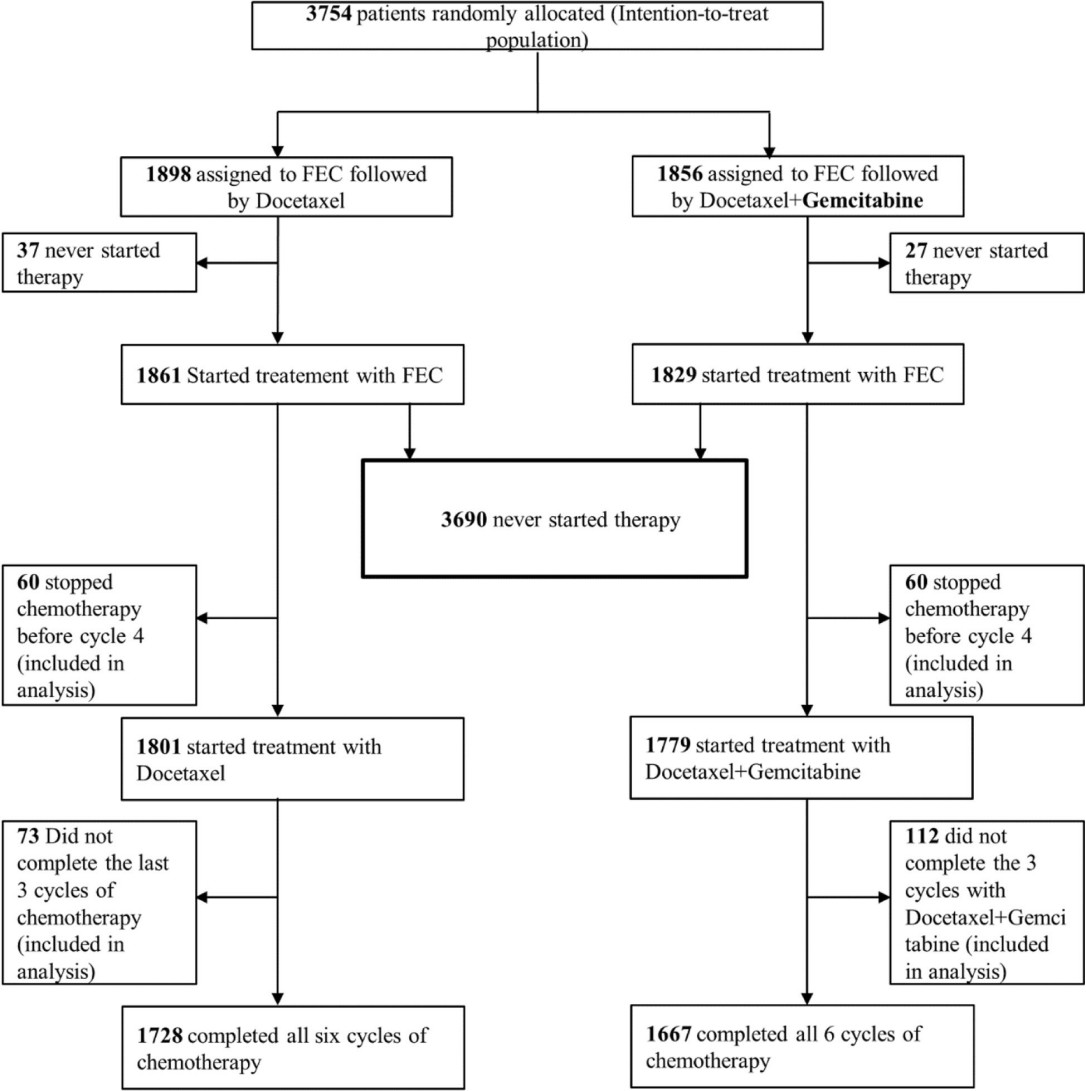
Patient flow chart (Consolidated Standards of Reporting Trials/CONSORT diagram)

From 2005–2007, 3754 patients from 251 study centers were randomly allocated to treatment groups (1898 FEC → Doc, 1856 FEC → Doc/Gem). The primary intention-to-treat analysis included all randomized patients. Sixty-four patients (37 FEC → Doc, 27 FEC → Doc/Gem) never started chemotherapy. A total of 3395 (90.4%) patients completed six cycles of chemotherapy, with 1728 (91.0%) receiving FEC → Doc and 1667 (89.9%) FEC → Doc/Gem. After three initial cycles of FEC, 1779 (94.9%) patients in the FEC → Doc/Gem arm started treatment with Doc/Gem and 1801 (95.9%) patients in the FEC → Doc arm started Doc monotherapy (Fig. 1).

Dose reductions and delays were necessary in 261 (13.8%) and 40 (2.1%) patients in the FEC → Doc and in 427 (23.0%) and 95 (5.1%) patients in the FEC → Doc/Gem group, respectively.

Baseline characteristics were complete for 97.3% of patients. Missing values for each variable were below 1.0%, with the exception of HER2 at 2.2%. Table 1 lists the patient characteristics in each treatment arm. The median follow-up periods for DFS were 5.2 (FEC → Doc) and 5.3 years (FEC → Doc/Gem), while for OS it was 5.3 years in both arms. Overall, 269 patients died during the study, 140 in the FEC → Doc and 129 in the FEC → Doc/Gem group. The numbers of patients with distant metastases in the two groups were 183 and 165, respectively, while corresponding numbers of local recurrences were 51 and 45, respectively, resulting in total numbers of DFS events of 239 and 219, respectively.

**Table 1:** Patient characteristics in each treatment arm

Characteristic	FEC → Doc		FEC → Doc/Gem	
	Mean or *n*	SD or %	Mean or *n*	SD or %
Age	53.9	10.4	53.1	10.6
BMI	26.3	5.1	26.2	5.
Tumor stage				
pT1	771	40.6	781	42.1
pT2	992	52.3	960	51.7
pT3	109	5.7	89	4.8
pT4	26	1.4	26	1.4
Grade				
G1	79	4.2	97	5.2
G2	914	48.2	891	48.0
G3	905	47.7	868	46.8
Nodal status				
pN+	1264	66.6	1217	65.6
pN0	634	33.4	639	34.4
Tumor type				
Ductal	1558	82.1	1524	82.1
Lobular	213	11.2	206	11.1
Other	127	6.7	126	6.8
ER				
Negative	610	32.1	642	34.6
Positive	1288	67.9	1214	65.4
PR				
Negative	768	40.5	757	40.8
Positive	1130	59.5	1099	59.2
HER2				
Negative	1457	76.8	1413	76.1
Positive	441	23.2	443	23.9
Menopausal status				
Premenopausal	775	40.8	790	42.6
Postmenopausal	1123	59.2	1066	57.4
Adjuvant antihormonal therapy	1360	72.3	1317	71.4
Adjuvant trastuzumab therapy	378	20.1	376	20.4
Adjuvant radiotherapy	1601	85.1	1553	84.2

*BMI* body mass index, *Doc* docetaxel, *ER* estrogen receptor, *FEC* 5-fluoroucacil, epirubicin, and cyclophosphamide, *Gem* gemcitabine, *HER2* human epidermal growth factor receptor 2, *PR* progesterone receptor, *SD* standard deviation

### Main analysis

**Fig. 2:**
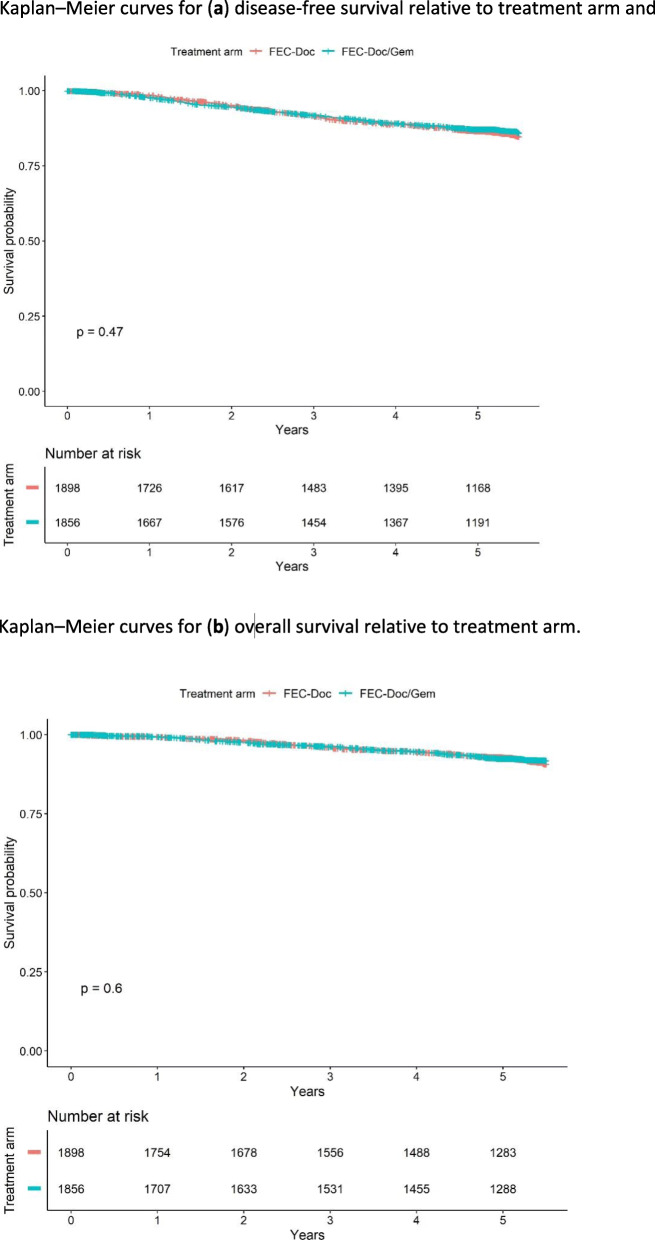
Kaplan–Meier curves for **a** disease-free survival relative to treatment arm and Kaplan–Meier curves for **b** overall survival relative to treatment arm

The study did not show any significant differences between the treatment arms, neither regarding DFS (HR = 0.93; 95%CI, 0.78–1.12; *P* = 0.47) nor OS (HR = 0.94; 95%CI, 0.74–1.19; *P* = 0.60); HR not adjusted. The 5-year DFS rates were 86.6% and 87.2% and the OS rates 92.8% and 92.5% in the FEC → Doc and FEC → Doc/Gem arms, respectively (see Table 2 and Fig. 2). In addition, no differences between the treatment arms were observed either for distant metastasis-free survival (HR = 0.92; 95%CI, 0.74–1.13; *P* = 0.43) or BC-specific survival (HR = 0.92; 95%CI, 0.71–1.20; *P* = 0.55).

**Table 2:** Numbers of events and 2-year and 5-year disease-free survival (DFS) and overall survival (OS) relative to treatment arm (95% confidence intervals in brackets)

	FEC → Doc	FEC → Doc/Gem
DFS		
Events	239	219
2-year rate	0.95 (0.94, 0.96)	0.95 (0.94, 0.96)
5-year rate	0.87 (0.85, 0.88)	0.87 (0.86, 0.89)
OS		
Events	140	129
2-year rate	0.98 (0.97, 0.99)	0.98 (0.97, 0.98)
5-year rate	0.93 (0.92, 0.94)	0.93 (0.91, 0.94)

### Further analyses

**Fig. 3:**
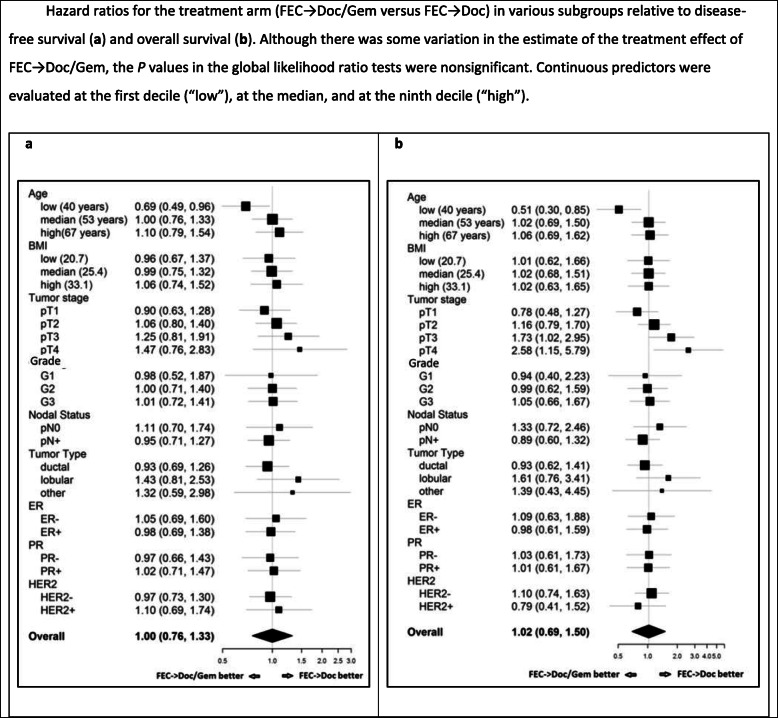
Hazard ratios for the treatment arm (FEC → Doc/Gem versus FEC → Doc) in various subgroups relative to disease-free survival (**a**) and overall survival (**b**). Although there was some variation in the estimate of the treatment effect of FEC → Doc/Gem, the *P* values in the global likelihood ratio tests were nonsignificant. Continuous predictors were evaluated at the first decile (“low”), at the median, and at the ninth decile (“high”)

Additional analyses taking into account well-known predictors for survival showed that no treatment effects were evident, neither for all patients nor within any specific subgroups of patients (*P* = 0.41 for DFS, *P* = 0.06 for OS; likelihood ratio tests). Although global likelihood ratio tests were not significant (and therefore interaction tests were not performed; see “Statistical methods”), there was some variation in the subgroups in the estimate of the treatment effect (subgroup-specific hazard ratios are shown in Fig. 3a for DFS and Fig. 3b for OS).

### Safety

Adverse events (AEs) occurred in 1803 (96.9%) of 1861 patients receiving FEC → Doc and 1800 (98.4%) of 1829 patients treated with FEC → Doc/Gem. Serious AEs occurred in 664 (35.8%) of the patients on FEC → Doc and 706 (37.2%) of those on FEC → Doc/Gem. AEs leading to treatment discontinuation occurred in 53 (2.8%) of the FEC → Doc and 67 (3.7%) of the FEC → Doc/Gem patients. The most common AEs in FEC treatment (cycles 1–3, both groups, all grades) were leukopenia (67.0%), alopecia (65.4%), and nausea (53.6%). Leukopenia (39.1%) and neutropenia (27.8%) were the most common grade 3 or 4 AEs during cycles 1–3. During cycles 4–6, leukopenia and anemia were the most common AEs in both groups (Doc and Doc/Gem). Granulocyte-colony stimulating factor (G-CSF) support was received by 24.3% of patients in the FEC → Doc group and by 23.4% of patients in the FEC → Doc/Gem group during the first three cycles. The corresponding values for the second three cycles were 36.3% and 57.8%. Grade 3 or 4 AEs that occurred more often with Doc/Gem than with Doc were leukopenia, nausea, anemia, serum glutamate pyruvate transaminase (SGPT) elevation, fatigue, and thrombopenia [22]. More detailed information on toxicity with extensive table for grade 3/4 AEs, information on death during the trial, long-term safety, use of G-CSF, and patient-reported quality of life are already published separately [22, 23].

## Discussion

This randomized phase III trial did not demonstrate any improvement in outcome with the addition of gemcitabine to FEC → Doc for early BC. DFS and OS were almost identical in the randomization arms, even in subgroup analyses, but toxicity was significantly higher in the FEC → Doc/Gem arm [22]. Thus, addition of gemcitabine does not contribute to further improvement of adjuvant cytotoxic treatment in those patients.

Regarding the dose of gemcitabine used in SUCCESS-A, we do not assume any negative impact by the chosen Gem-dosis on the presented outcome as the results of SUCCESS-A are complementary to the adjuvant and neoadjuvant studies tAnGo, Neo-tAnGo, NSAPB-B38, and NSABP-B40 testing gemcitabine [24–28]. It should be noted that Neo-tAnGo and NSABP-B38 used the highest dose intensity (1000 mg/m^2^/week) [26, 28], while tAnGo had a dose intensity of 833 mg/m^2^/week [27] and NSABP-B40 and SUCCESS-A used a dose intensity of 666 mg/m^2^/week [24, 25]. Although it might be hypothesized that these relevant differences in gemcitabine dose intensity may influence efficacy, there are two studies at each end of the dose intensity range (Neo-tAnGo/NSABP-B38 with a high and NSABP-B40/SUCCESS-A with a low dose) uniformly showing no improvement in the prognosis with either application scheme or dose [24, 26, 28]. While these results do not support the use of Gemcitabine in the (neo)adjuvant setting, the observed phenomenon is already known as several agents showing efficacy in the metastatic setting failed to become established in treatment for early BC like capecitabine [29–31] or lapatinib [32].

Though this study prospectively randomized more than 3700 patients within a national phase III trial and thereby is one of the largest performed clinical trial for breast cancer in Germany ever, limitations need to be clearly addressed. As the number of expected events was much higher than the number of observed events, the study is formally underpowered. However, as the presented results are unambiguous, we do not expect any relevant bias due to the lower rate of events. As breast cancer follow-up usually consists of 10 years due to well-known late recurrences, the study follow-up with only 5 years is quite short and so a potentially relevant proportion of disease recurrences might have been missed due to their later appearance. Furthermore, the definition of DFS used for the SUCCESS-A trial including contralateral invasive disease recurrence (according to the STEEP system [20]) might affect the final interpretation, as these recurrences might represent new primary cancers and not a recurrence of the initially treated cancer. The non-subtype based study design with inclusion of more than 3000 patients does not represent the current trend in oncology any more. More recent clinical trials for improvement of cancer therapy usually include patients with well-defined biological subtypes and predictive biomarkers such as genomics that potentially can identify patients who may benefit from a given treatment. Furthermore, none of the stratification factors was a pre-specified factor powered to show a difference in hazard ratio. 

In addition, also some data published after the start of the SUCCESS-A study affect the interpretation of the present results in terms of new standard chemotherapy regimens. Although FEC → Doc is regarded as a highly active chemotherapy regimen in high-risk BC patients, using 5-FU may not be necessary in the adjuvant treatment of BC. During the development of chemotherapies, FEC was introduced by replacing methotrexate in the commonly used CMF scheme [33], and later, the omission of 5-FU was tested [34]. In the GIM-2 study with node-positive patients, adding 5-FU to a regimen with epirubicin and cyclophosphamide followed by paclitaxel did not improve the DFS [34]. In clinical practice, weekly paclitaxel may also be used more often than three-weekly docetaxel as weekly paclitaxel results in a longer OS than using either paclitaxel or docetaxel every 3 weeks or docetaxel weekly [35]. Although FEC → Doc is not the preferred choice of chemotherapy in the adjuvant situation anymore, the used chemotherapy backbone in SUCCESS-A consisting of an anthracycline-taxane-containing chemotherapy regimen is still standard of care in breast cancer treatment. The present study—along with the NSAPB-B38/40 and tAnGo/Neo-tAnGo trials—clearly provides solid evidence that adding gemcitabine to chemotherapy in the adjuvant setting does not improve the prognosis for patients with BC.

Further progress in systematic treatment of oncological patients will unlikely come from large-scale phase III studies including thousands of patients. Due to high toxicity with potentially severe long-term morbidity, use of chemotherapy and also number of applicated cytotoxic agents should be reduced and restricted to those patients who clearly benefit from those therapies. Molecular tests based on multigene expression profiling indicated that a large proportion of patients might not need chemotherapy at all [1, 2], and further research is focused on identification of high-risk patients and of those patient groups most likely to benefit from a specific treatment.

However, as long as there are no specific molecular profiles or predictive factors for gemcitabine-related efficacy identified, gemcitabine is not used for the overall population of adjuvant high-risk BC patients. With the presented results of SUCCESS-A, the role of gemcitabine as an added agent in adjuvant chemotherapy regimen is clearly and finally determined, as the addition of gemcitabine did not show improved survival outcomes, but was associated with increased toxicity.

## Conclusion

Within the phase III SUCCESS-A trial, 3754 high-risk early breast cancer patients were randomized for adjuvant chemotherapy. Thereby, the addition of gemcitabine to standard chemotherapy (FEC/DOC) was analyzed. Adding gemcitabine to a standard chemotherapy does not improve the outcomes in patients with high-risk early breast cancer and should therefore not be included in the adjuvant treatment setting.

## Data Availability

The datasets used and/or analyzed during the current study are available from the corresponding author on reasonable request.

## Appendix A

### Full inclusion and exclusion criteria

**Table:**

*Inclusion criteria*	
Patients may be included in the study only if they meet all the following criteria:	
1	Primary epithelial invasive carcinoma of the breast pT1–4, pM0
2	Histopathological confirmation of axillary lymph node metastases (pN1–3) or high-risk pN0/NX, defined as: “pT ≥ 2 or histopathological grade 3 or age ≤ 35 or negative hormone receptor status”
3	Complete resection of the primary tumor, with resection margins free of invasive carcinoma, no more than 6 weeks previously
4	Females ≥ 18 years of age
5	Performance status ≤ 2 on the Eastern Cooperative Oncology Group (ECOG) scale
6	Adequate bone marrow reserve: leukocytes ≥ 3.0 × 10^9^/L and platelets ≥ 100 × 10^9^/L
7	Bilirubin within 1-fold of the reference laboratory’s normal range, ASAT (SGOT), ALAT (SGPT), and AP within 1.5-fold of the reference laboratory’s normal range for patients
8	Intention to attend regular follow-up visits for the duration of the study
9	Ability to understand the nature of the study and to provide written informed consent
*Exclusion criteria*	
Patients will be excluded from the study for any of the following reasons:	
10	Inflammatory breast cancer
11	Previous or concomitant cytotoxic or other systemic antineoplastic treatment that is not part of or not allowed in this study
12	History of treatment or disease affecting bone metabolism (e.g., Paget’s disease, primary hyperparathyroidism)
13	Prior treatment with bisphosphonates within the previous 6 months
14	Severe renal insufficiency as evidenced by creatinine clearance (CrCl) < 30 mL/min, as calculated using the Cockcroft–Gault formula: $\mathrm{CrCl}=\frac{140-\mathrm{age}\left(\mathrm{years}\right)\times \mathrm{weight}\ \left(\mathrm{kg}\right)\times 0.85}{72\times \mathrm{serum}\ \mathrm{creatinine}\ \left(\frac{\mathrm{mg}}{\mathrm{dl}}\right)}$
15	Second primary malignancy (except in situ carcinoma of the cervix or adequately treated basal cell carcinoma of the skin)
16	Cardiomyopathy with impaired ventricular function (New York Heart Association > II), cardiac arrhythmias influencing left ventricular ejection fraction and requiring medication, history of myocardial infarction or angina pectoris within the previous 6 months, or arterial hypertension not controlled by medication
17	Any known hypersensitivity against docetaxel, epirubicin, cyclophosphamide, fluorouracil, gemcitabine, or any other medication included in the study protocol
18	Use of any investigational agent within 3 weeks prior to inclusion
19	Patients in pregnancy or breastfeeding (in premenopausal women contraception has to be ensured: intrauterine devices, surgical sterilization methods, or—in hormone-insensitive tumors only—oral, subcutaneous or transvaginal hormonal, non-estrogen-containing contraceptives)
20	Current active dental problems, including infection of the teeth or jaw (maxilla or mandible); dental or fixture trauma, or a current or prior diagnosis of osteonecrosis of the jaw (ONJ), of exposed bone in the mouth, or of slow healing after dental procedures
21	Recent (within 6 weeks) or planned dental or jaw surgery (e.g., extraction, implants)

## Appendix B

### Dose-level adjustments

Treatment could be postponed for up to 2 weeks if the patient had not recovered from hematological and/or nonhematological toxicity. Primary prophylactic application of granulocyte-colony stimulating factor (G-CSF) was not recommended unless one of the following conditions applied: previous febrile neutropenia, prolonged neutropenia for more than 5 days with an absolute neutrophil count (ANC) < 0.5 × 10^9^/L, ANC < 0.1 × 10^9^/L, or prolongation of a chemotherapy cycle due to insufficient leukocytes or neutrophils. Dose reduction was performed in accordance with a prespecified dose level table^a^:

**Table:**

	Dose level		
	0	− 1	− 2
Fluorouracil	500 mg/m^2^	400 mg/m^2^	300 mg/m^2^
Epirubicin	100 mg/m^2^	80 mg/m^2^	60 mg/m^2^
Cyclophosphamide	500 mg/m^2^	400 mg/m^2^	300 mg/m^2^
Docetaxel in combination with gemcitabine	75 mg/m^2^	60 mg/m^2^	45 mg/m^2^
Docetaxel as monotherapy	100 mg/m^2^	80 mg/m^2^	60 mg/m^2^
Gemcitabine (each arm)	1000 mg/m^2^	800 mg/m^2^	600 mg/m^2^

^a^With regard to hematological toxicity, dose levels were subsequently reduced by one level if the patient had an ANC of < 0.5 × 10^9^/L, febrile neutropenia, or prolongation of the chemotherapy cycle despite G-CSF use. Dose levels were also to be adjusted subsequently by one dose level if the patients developed grade 4 thrombocytopenia. Grade 2 neurological toxicities led to a reduction by one dose level, and grade 3/4 toxicity resulted in termination of treatment. Grade 3 gastrointestinal toxicity resulted in a reduction by one level and grade 4 led to termination of treatment. Cardiac events such as arrhythmia that required treatment, arteriovenous occlusion more severe than grade 1, and a reduction of the left ventricular ejection fraction below the normal clinical range also resulted in termination of treatment. Grade 2 or higher pneumonitis related to gemcitabine resulted in permanent discontinuation of the chemotherapy. Any other form of grade 4 toxicity, or prolongation of any chemotherapy cycle by more than 2 weeks, also led to treatment termination

## Abbreviations

AE: Adverse event
ANC: Absolute neutrophil count
BC: Breast cancer
BMI: Body mass index
CI: Confidence interval
CMF: Cyclophosphamide, methotrexate, and fluorouracil
CTC: Circulating tumor cells
DFS: Disease-free survival
Doc: Docetaxel
ER: Estrogen receptor
FEC: 5-Fluoroucacil, epirubicin and cyclophosphamide
FEC-Doc: 5-Fluorouracile, epirubicin, cyclophosphamide-docetaxel
FEC-Doc/Gem: 5-Fluorouracile, epirubicin, cyclophosphamide-docetaxel, gemcitabine
5-FU: 5-Fluorouracil
G-CSF: Granulocyte-colony stimulating factor
Gem: Gemcitabine
HER2: Human epidermal growth factor receptor 2
HR: Hazard ratio
HRS: Hormone receptor status
OS: Overall survival
PR: Progesterone receptor
SGPT: Serum glutamate pyruvate transaminase
